# Renal dysfunction as a predictor of recurrence and prognosis in colorectal cancer

**DOI:** 10.3389/fonc.2025.1606286

**Published:** 2025-06-27

**Authors:** Xuekang Ren, Shaogong Zhu, Rongzhen Li, Yunzhan Xia

**Affiliations:** Department of General Surgery, The Fifth Clinical Medical College of Henan University of Traditional Chinese Medicine, Zhengzhou, Henan, China

**Keywords:** colorectal cancer, kidney function, EGFR, recurrence, clinical prognosis

## Abstract

**Purpose:**

This study explores the relationship between eGFR and recurrence and prognosis in patients with colorectal cancer (CRC).

**Methods:**

Patients first diagnosed with CRC at Zhengzhou People’s Hospital between 2018 and 2021 with a median follow-up of 715 days were studied. Demographics, disease characteristics, kidney function were collected. Associations between eGFR and clinical prognosis were assessed using multivariate Cox proportional hazards regression models. The impact of eGFR on the recurrence was evaluated by logistic and Poisson regression models. Odds ratios are reported for associations between eGFR and recurrence and prognosis. Stratified analyses and restricted cubic splines (RCS) were used to evaluate the results between subgroups and nonlinear relation between eGFR and the prognosis and recurrence of patients with CRC.

**Results:**

93 CRC patients completed the study. Poor renal filtration function and impaired urine concentrating ability were found in CRC patients. Multivariate analysis showed that eGFR was an independent predictor of clinical prognosis (eGFR < 90 mL/min OR=4.248, 95% CI [1.061-17.003], *P*=0.041, the eGFR of 90–110 mL/min OR=5.087, 95% CI [1.268-20.400], *P*=0.022) when using the eGFR ≥ 130 mL/min as the reference. Multivariate analysis showed that eGFR (OR=0.964, 95% CI [0.933-0.997], *P*=0.032) was an independent protective factor influencing recurrence of CRC patients. RCS analysis showed that the relationship between eGFR and prognosis of CRC patients had no significant nonlinear correlation (*P* for nonlinear=0.19), the relationship between eGFR and recurrence was non-linear (*P* for nonlinear<0.001).

**Conclusions:**

CRC patients exhibited kidney dysfunction, and eGFR is identified as an independent predictor of disease recurrence and prognosis.

## Introduction

Colorectal cancer (CRC) is the third most prevalent gastrointestinal malignancy worldwide (1, 2). It is characterized by a high rate of recrudescence and mortality, imposing a great burden on the family and society (3, 4). Hence, more potential biomarkers should be included in clinical practice to improve prognostic prediction.

In fact, the development and outcome of CRC are influenced by various genetic and environmental risk factors (5). Previous studies have revealed that chronic inflammatory state correlates with a bad patient prognosis and poor clinical outcome of CRC (6, 7). Studies have shown that elevated IL-6 (8), systemic immune-inflammation index (9), neutrophil-lymphocyte ratio (10), and lymphocyte ratio are all prognostic markers for poor outcome of CRC patients (11). Despite the availability of these prognostic markers, high-risk CRC patients cannot be identified with sufficient accuracy.

Consistently, renal insufficiency often represents a state of chronic inflammatory stress (12), and clinical observations of patients with CRC with renal dysfunction suggest a possible association between the two diseases. Recently, the standardized incidence ratio (SIR) of CRC was obtained from 14 studies and showed a significant relationship between CRC with chronic kidney disease (CKD) patients, with a pooled SIR of 1.33, which indicates that patients with CKD have a significantly increased risk of CRC (13). And in patients with CKD, cancer is the second leading cause of death after cardiovascular diseases, especially among those with end-stage renal disease (14). Nevertheless, the relationship between renal function and clinical prognosis of CRC patients remains unexplored.

Renal dysfunction was associated with various disorders, such as metabolic (15), autoimmune (16), and tumor-related diseases (17). Upon kidney dysfunction, some toxic agents may promote chronic inflammation generating oxidative stress-induced DNA damage that may activate tumor-promoting genes and inactivate tumor-suppressing genes (18, 19). Conversely, DNA damage might also lead to inflammation and promote tumorigenesis (20). Therefore, renal insufficiency may be closely related to the occurrence and development of tumors.

Since CRC patients are often diagnosed at an advanced stage, as there are no obvious symptoms in the early stages of the disease, and no sensitive markers for disease recurrence and prognosis, in view of the above, we speculated whether there would be early changes in renal function in patients with CRC and investigate the link between kidney function and risk for subsequent recurrence and prognosis in patients with CRC.

Estimated Glomerular filtration rate (eGFR) is the best indicator of kidney function. Therefore, we investigated and compared the kidney function indexes in patients with CRC and healthy controls (HCs) and evaluated clinical significance and prognostic value of eGFR in patients with CRC who were first pathologically diagnosed with CRC and received radical surgery or initial chemotherapy. In addition, we tested whether higher level of eGFR is associated with better prognosis and lower recurrence rate of CRC. It is of significance to identify novel prognostic marker for CRC and in hope of seeking novel treatment direction.

## Methods and materials

### Study population

The retrospective cohort study was performed at a tertiary university hospital serving a population of more than 20,000,000 each year in Central China. In the current study, we consecutively included patients with CRC from the hospital-based cohorts of Zhengzhou People’s hospital between 2018 and 2021. The median follow-up period was 715 (interquartile range [IQR] 356-978) days. Participants who fulfilled the inclusion criteria and none of the exclusion criteria were invited to participate in the study. Inclusion criteria are as follows: (1) all patients were first pathologically diagnosed with CRC; (2) the diagnosis was referenced to the latest (8th edition) American Joint Committee on Cancer (AJCC) CRC staging system; (3) patients who underwent radical surgery or received chemotherapy; (4) all patients had complete clinical information. The exclusion criteria were as follows: (1) age < 18 years; (2) with a previous CRC diagnosis and inability to consent; (3) undergoing chronic dialysis or having a kidney transplant or other kidney diseases; (4) combined with other types of tumors; (5) patients with a follow-up period shorter than 3 months.

After applying the inclusion and exclusion criteria, a total of 93 CRC patients were included in this study (**Supplementary Figure S1**). 81 healthy controls (HCs) were recruited through the Physical Examination Center of Zhengzhou People’s hospital. They were in good health as assessed by physical exam, electrocardiogram, and laboratory testing including routine blood and urine tests, liver, and kidney function tests.

### Data collection

Observational data of patients on admission were retrospectively collected from our hospital electronic medical records. The detailed data collection consisted of demographic characteristics, comorbidities (mainly including hypertension, diabetes, and coronary heart disease). In addition, the patients’ smoking history, drinking history, and current and past medical history were collected. Moreover, the presenting symptoms, hospitalization data, ancillary examinations (mainly including kidney function, routine urine examinations) were also collected. All indicators of kidney function were obtained before treatment and any type of intervention. Besides those, the pathological data, including tumor location, size, lymphatic metastasis, distant metastasis, perineural invasion, pathological pattern, tumor differentiation degree, and TNM stage were also collected. The decisions about the choice of surgical methods and treatment duration were based on physicians’ experience. All data were checked by two attending general surgeons.

### Covariates

Covariates were defined at baseline included onset age, sex, comorbid conditions, kidney function indexes, smoking history, drinking history, tumor location, size, lymphatic metastasis, distant metastasis, perineural invasion, pathological pattern, tumor differentiation degree, and TNM stage.

### Definitions

Recurrence was defined as recurrence of tumor after receiving radical surgery or initial chemotherapy (21). All the recurrence included local recurrence (recurrence in the residual or para-intestinal area), regional (recurrence in abdominal lymph nodes), and distant (recurrence in the area outside the intestinal tract including paraaortic lymph nodes, distant organs, and peritoneal cavity). The poor clinical prognosis was defined as death of the patients during the period of follow-up. We confirmed that all methods were performed in accordance with the relevant guidelines and regulations. The outcome variable was a binary variable of recurrence or not and death or not.

### Follow-up and outcome assessment

Patients were followed up every 3 months for the first 3 years, every 6 months in years 4 to 5, and at 12-month intervals after this time. We used chest x-rays and either computed tomography, magnetic resonance imaging, or positron emission tomography scan once a year and at any suspicion of recurrence based on bimanual examination, symptoms, and an increase in serum tumor marker that was elevated preoperatively. Overall survival included death from any cause. The follow-up period was uniformly defined as the time from surgery until death or, if the patient survived, the time from surgery until the last confirmation by personal visit, telephone contact, or through the nearby affiliated hospital. The Strengthening the Reporting of Observational Studies in Epidemiology (STROBE) reporting guideline was followed.

### Statistical analysis

GraphPad Prism 8.0 software (GraphPad Software Inc, San Diego, CA, USA), SPSS Statistics 24.0 (IBM, Armonk, NY, USA) and The R language software (R-4.1.0) were used for statistical analyses and graphing. The observed clinical and demographic continuous data were expressed by means (Standard deviation, SD), median (IQR), while categorical data and discontinuous variables were expressed by frequencies, and percentages. The data obtained in this study were tested for normality. The Mann-Whitney U tests were used to compare demographic factors and clinical characteristics. Pearson’s chi-squared or Fisher’s exact test was used for categorical variables where applicable. Student’s t test was used to compare kidney function indexes between patients with CRC and HCs. The Spearman correlation was used to test the association of eGFR and TNM stage in CRC patients. Additionally, ROC curve analysis was used to evaluate the diagnostic capacity of eGFR in distinguishing recurrence and no- recurrence, death and survival in patients with CRC. The survival curve for each group was plotted through the Kaplan-Meier survival analysis, and we also calculated incidence rates with 95% confidence interval (CI) using the exact method. A multivariate Cox regression analysis was performed by incorporating significant factors in the univariate Kaplan-Meier model to evaluate the factors affecting the prognosis. In a logistic regression analysis, recurrence was taken as dependent variable. Logistic regression models were used to assess whether eGFR or other covariates had an independent effect on the recurrence, and multivariate logistic regression models were used to analyze the independent predictors on the recurrence. Last, using the rms R package, Restricted Cubic Splines (RCS) were used to examine the relationship between the eGFR and survival outcomes or recurrence after adjusting for potential confounding factors including tumor differentiation degree, distant metastasis, lymphatic metastasis, and TNM stage. Differences were considered significant when the *P* value was less than 5% (*P*< 0.05).

## Results

### Baseline characteristics

There were 135 consecutive patients with CRC, 291 HCs accessing health care and undergoing kidney function testing and routine urine examinations between 2018 and 2021 in Zhengzhou People’s hospital. Following the inclusion and exclusion criteria, 93 participants with CRC and 81 HCs completed the study (**Supplementary Figure S1**). And the baseline characteristics between included and excluded patients with CRC are shown in **Supplementary Table 1** (**Supplementary Table S1**). The mean onset age of participants with CRC was 58.45 (SD=7.73), and 38.71% were women (**Table 1**) and the median duration of follow-up was 715 (IQR 356-978) days. The mean eGFR was 109.30 mL/min (SD=16.36) for the CRC patients; 16.13% of CRC patients had an eGFR ≥ 130 mL/min, 38.71% had an eGFR between 110 and 130 mL/min, 23.66% had an eGFR between 90 and 110 mL/min, 21.5% had an eGFR < 90 mL/min (**Table 1**). In addition, we found that distant metastasis and low tumor differentiation are more likely to occur in CRC patients with the eGFR < 90 mL/min. The specific baseline characteristics of CRC patients according to eGFR stratification were shown in **Table 1**. The results for the baseline characteristics of HCs and patients of CRC are reported in the **Supplementary Table 2** (**Supplementary Table S2**).

**Table 1:** Baseline characteristics of CRC overall and by eGFR categories.

Basic Information		eGFR Strata, mL/min				
	Overall	eGFR < 90	eGFR 90-110	eGFR 110-130	eGFR ≥ 130	P Value
Patient numbers no. (%)	93	20 (21.50)	22 (23.66)	36 (38.71)	15 (16.13)	–
Clinical characteristics						
eGFR, mean (SD), mL/min	109.30 (16.36)	87.34 (3.03)	101.44 (8.74)	116.47 (6.67)	132.88 (2.59)	**<0.001**
Female, no. (%)	36 (38.71)	9 (45.00)	9 (40.91)	12 (33.33)	6 (40.00)	0.843
Onset age, mean (SD), years	58.45 (7.73)	59.73 (8.44)	57.73 (5.96)	57.72 (9.31)	59.57 (4.55)	0.701
Follow-up time, median (IQR), days	715 (356-978)	726 (404-949)	399 (296-724)	668 (345-872)	1212 (393-876)	**<0.001**
Recurrence, no. (%)	50 (53.76)	15 (75.00)	14 (63.64)	19 (52.78)	2 (13.33)	**0.002**
Deaths, no. (%)	42 (45.16)	17 (85.00)	12 (54.55)	10 (27.78)	3 (20.00)	**<0.001**
Comorbidity						
Hypertension, no. (%)	20 (21.51)	5 (25.00)	5 (22.73)	7 (19.44)	3 (20.00)	0.965
Diabetes, no. (%)	16 (17.20)	3 (15.00)	8 (36.36)	5 (13.89)	0 (0)	**0.028**
Coronary heart disease, no. (%)	14 (15.05)	4 (20.00)	0 (0)	8 (22.22)	2 (13.33)	0.124
Personal history						
Smoke, no. (%)	24 (25.81)	5 (25.00)	6 (27.27)	13 (36.11)	0 (0)	0.065
Drink, no. (%)	35 (37.63)	8 (40.00)	5 (22.73)	13 (36.11)	9 (60.00)	0.147
Tumor characteristics						
Rectum, no. (%)	50 (53.76)	8 (40.00)	15 (68.18)	18 (50.00)	9 (60.00)	0.283
Tumor size<4cm, no. (%)	64 (68.82)	11 (55.00)	14 (63.64)	26 (72.22)	13 (86.67)	0.214
Lymphatic metastasis, no. (%)	22 (23.66)	7 (35.00)	6 (27.27)	8 (22.22)	1 (6.67)	0.259
Distant metastasis, no. (%)	33 (35.48)	12 (60.00)	9 (40.91)	9 (25.00)	3 (20.00)	**0.032**
Perineural invasion, no. (%)	10 (10.75)	2 (10.00)	2 (9.09)	5 (13.89)	1 (6.67)	0.872
Adenocarcinoma, no. (%)	76 (81.72)	15 (75.00)	20 (90 ().91)	28 (77.78)	13 (86.67)	0.481
Low differentiation, no. (%)	29 (31.18)	16 (80.00)	9 (40.91)	3 (8.33)	1 (6.67)	**<0.001**
TNM, I stage, no. (%)	32 (34.41)	4 (20.00)	9 (40.91)	11 (30.56)	8 (53.33)	0.182

eGFR, estimated glomerular filtration rate; SD, standard deviation; IQR, interquartile range; Bold entries indicate *P* < 0.05. TNM, tumor node metastasis classification.

### Kidney dysfunction in patients with CRC

**Figure 1:**
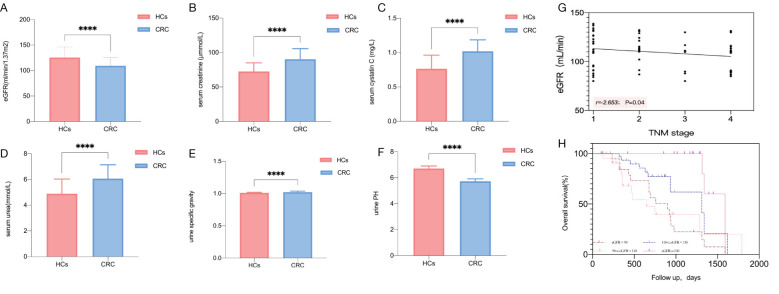
Comparison of renal function parameters between CRC patients and HCs. Comparison of eGFR **(A)**, serum creatinine concentration **(B)**, serum cystatin C **(C)**, serum urea concentration **(D)**, urine specific gravity **(E)**, urine PH **(F)**. Correlation plots of eGFR and tumor TNM stage **(G)**. The time to death by eGFR stratification as assessed with the Kaplan-Meier estimation. Log-rank (Mantel-Cox) test *P* < 0.001 **(H)**. Statistical significance is defined by *P <*0.05. **** *P* < 0.0001.

The overall results of the kidney function assessment in this study of patients with CRC and HCs are shown in **Figure 1**. We found that eGFR was significantly lower in patients with CRC than that in HCs (*P* < 0.001) (**Figure 1A**). The serum creatinine (**Figure 1B**), serum cystatin C (**Figure 1C**), serum urea (**Figure 1D**), and urine specific gravity (**Figures 1E**) was significantly higher in patients with CRC than those in HCs (*P* < 0.001), respectively. Additionally, urine PH (**Figure 1F**) in CRC patients was significantly lower than that in HCs (*P* < 0.001). In addition, we found that eGFR is negatively correlated with the TNM stage (*r*=-2.653, *P* = 0.04) (**Figure 1G**).

### Increased risks of death and recurrence in CRC patients with decreased eGFR

Over a median follow-up of 715 days, 50 cases of CRC patients with recurrence were identified. The baseline characteristics of relapsing and no relapsing CRC patients are shown in **Supplementary Table 3** (**Supplementary Table S3**). The highest mortality risk was noted for CRC patients with eGFR < 90 mL/min (1.12 per 1,000 person-days (pd) with eGFR < 90 mL/min, and the recurrence rate was 75%; 0.98 per 1,000 pd with eGFR 90–110 mL/min, and the recurrence rate was 63.6%; 0.42 per 1,000 pd with eGFR 110–130 mL/min, and the recurrence rate was 52.8%; 0.17 per 1,000 pd with eGFR ≥ 130 mL/min, and the recurrence rate was 13.3%) (**Supplementary Table S4**, **Supplementary Figure S3**). In addition to the above, 42 cases of CRC patients with death were identified. The baseline characteristics of dead and survival CRC patients are shown in **Supplementary Table 5** (**Supplementary Table S5**). The highest mortality incidence was noted for CRC patients with eGFR < 90 mL/min with a mortality rate of 85%, the lowest mortality incidence was noted for CRC patients with eGFR ≥ 130 mL/min with a mortality rate of 20% (**Supplementary Figure S3**). Furthermore, Kaplan-Meier curves indicated that there was a higher mortality incidence in CRC patients with lower eGFR categories (**Figure 1H**).

### eGFR as a predictor of clinical prognosis in patients with CRC

To further assess whether lower eGFR is a risk factor for poor prognosis in CRC patients, we conducted univariate and multivariate Cox regression analyses, defining poor clinical prognosis as death. Finally, 42 cases of death with poor clinical prognosis were identified, and the overall poor clinical prognosis incidence rate was 45.16%. Univariate analysis by cox regression models of factors affecting the clinical prognosis of patients with CRC showed that the eGFR<90 mL/min (odds ratio [OR] 7.804, 95% CI [2.273-26.791], *P*=0.001) and the eGFR of 90–110 mL/min (OR 6.997, 95% CI [1.912-25.608], *P*=0.003) were risk factors of poor clinical prognosis when using the eGFR ≥130 mL/min as the reference, while there was no significant difference with eGFR of 110–130 mL/min (**Table 2**). Multivariate analysis showed that eGFR was still an independent predictor of clinical prognosis. Moreover, similar results to those described above were found when eGFR was used as a continuous variable (**Supplementary Table S6**). In addition, we found that distant metastasis (OR 16.443, 95% CI [2.877-93.979], *P*=0.002) and TNM stage (OR 6.342, 95% CI [2.246-17.910], *P*<0.001) were also the risk factors of poor clinical prognosis (**Table 2**).

**Table 2:** Univariate and multivariable analysis of factors affecting the prognosis of patients with CRC by cox regression models.

Variable	Univariate analysis		Multivariate analysis	
	OR (95%CI)	*P* value	OR (95%CI)	*P* value
Clinical characteristics				
eGFR		**0.003**		**0.073**
≥130	REF	–	REF	–
110-130	3.334 (0.901-12.333)	0.071	2.017 (0.526-7.736)	0.306
90-110	6.997 (1.912-25.608)	**0.003**	5.087 (1.268-20.400)	**0.022**
<90	7.804 (2.273-26.791)	**0.001**	4.248 (1.061-17.003)	**0.041**
Sex	0.971 (0.521-1.809)	0.926		
Onset age	0.896 (0.484-1.657)	0.726		
Comorbidity				
Hypertension	0.788 (0.371-1.670)	0.534		
Diabetes	0.680 (0.320-1.444)	0.315		
Coronary heart disease	0.580 (0.251-1.340)	0.202		
Personal history				
Smoke	1.668 (0.765-3.637)	0.198		
Drink	1.136 (0.599-2.154)	0.696		
Tumor characteristics				
Location	0.900 (0.487-1.663)	0.736		
Tumor size	0.603 (0.298-1.221)	0.160		
Lymphatic metastasis	0.607 (0.317-1.160)	0.131		
Distant metastasis	3.344 (1.560-7.167)	**0.002**	16.443 (2.877-93.979)	**0.002**
Perineural invasion	1.692 (0.648-4.416)	0.283		
Pathological pattern	0.775 (0.353-1.700)	0.525		
Differentiation	2.561 (1.346-4.873)	**0.004**	1.280 (0.567-2.887)	0.552
TNM stage	2.081 (1.418-3.054)	**<0.001**	6.342 (2.246-17.910)	**<0.001**

eGFR, estimated glomerular filtration rate; REF, Reference; OR, Odds ratio; CI, confidence interval; TNM, tumor node metastasis classification; Bold entries indicate *P* < 0.05. eGFR was parameterized as a categorical variable.

### eGFR as a predictor of clinical recurrence in patients with CRC

To further evaluate whether the lower eGFR is an impact factor of the occurrence of recurrence in patients with CRC, we performed univariate and multivariate logistic regression analysis. Finally, 50 cases with recurrence were identified, and the overall rate of recurrence was 53.76%. Univariate analysis by logistic regression models of factors affecting the recurrence of patients with CRC showed that the eGFR (OR 0.966, 95% CI [0.942-0.991], *P*=0.007) was an independent protective factor influencing recurrence of CRC when it was used as a continuous variable (**Table 3**), and similar results were also seen when eGFR was used as a categorical variable (**Supplementary Table S7**). We then adjusted for the variables in multivariable logistic regression models. The findings revealed that the eGFR (OR 0.964, 95% CI [0.933-0.997], *P*=0.032) was still an independent protective factor influencing recurrence of CRC (**Table 3**). Moreover, using univariate Poisson regression model, we identified that eGFR (OR 0.968, 95% CI [0.955-0.981], *P*<0.001) was associated with a statistically significant 3.2% relative reduction in the number of recurrences in patients with CRC when eGFR was used as a continuous variable (**Supplementary Table S8**). Moreover, using multivariate Poisson regression model, we further defined that eGFR (OR 0.970, 95% CI [0.956-0.984], *P*<0.001) was independently associated with a decreased OR of counts of relapsing attacks, and we found that lymphatic metastasis (OR 1.644, 95% CI [1.107-2.442], *P*=0.014) was independently associated with an increased OR of counts of relapsing attacks (**Supplementary Table S8**).

**Table 3:** Univariate and multivariable analysis of factors affecting the recurrence of patients with CRC by logistic regression models.

Variable	Univariate analysis		Multivariate analysis	
	OR (95%CI)	*P* value	OR (95%CI)	*P* value
eGFR	0.966 (0.942-0.991)	**0.007**	0.964 (0.933-0.997)	**0.032**
Sex	1.537 (0.664-3.558)	0.316		
Onset age	2.083 (0.909-4.773)	0.083		
Hypertension	0.939 (0.347-2.535)	0.900		
Diabetes	0.886 (0.299-2.620)	0.827		
Coronary heart disease	1.194 (0.383-3.725)	0.759		
Smoke	0.779 (0.305-1.993)	0.603		
Drink	1.682 (0.722-3.919)	0.228		
Location	2.061 (0.899-4.725)	0.088		
Tumor size	0.494 (0.199-1.227)	0.129		
Lymphatic metastasis	5.484 (1.685-17.848)	**0.005**	3.515 (0.902-13.705)	0.070
Distant metastasis	5.571 (2.088-14.867)	**0.001**	3.351 (0.416-26.988)	0.256
Perineural invasion	1.865 (0.490-7.102)	0.361		
Pathological pattern	1.390 (0.484-3.988)	0.541		
Differentiation	5.253 (1.882-14.660)	**0.002**	1.321 (0.349-5.002)	0.682
TNM stage	1.852 (1.302-2.634)	**0.001**	0.991 (0.450-2.183)	0.983

eGFR, estimated glomerular filtration rate; OR, Odds ratio; CI, confidence interval; TNM, tumor node metastasis classification; Bold entries indicate *P* < 0.05. eGFR was parameterized as a continuous variable.

### Stratified analysis for the association of the eGFR with the outcome of recurrence and prognosis in CRC patients

**Figure 2:**
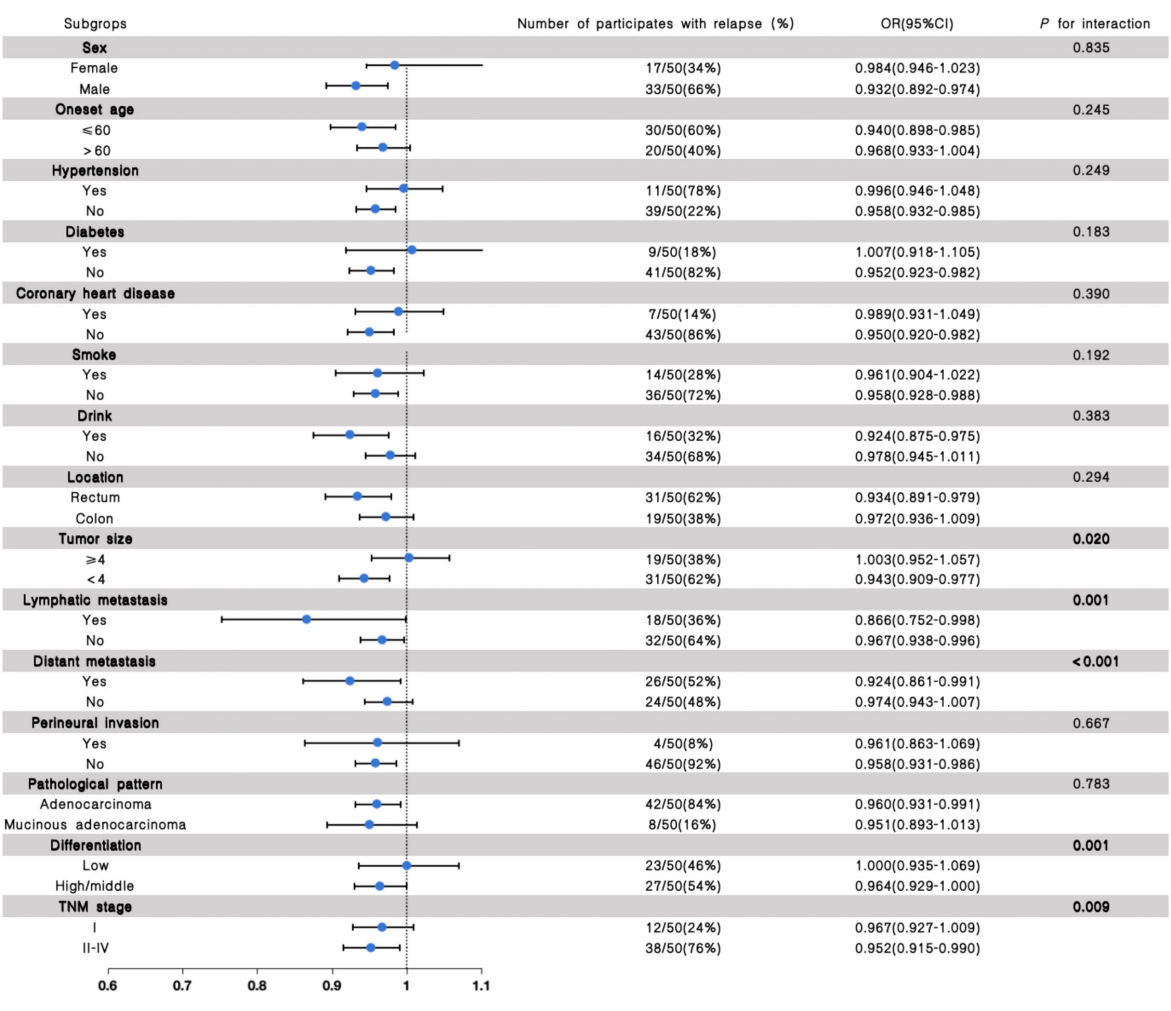
The results of the stratified and interaction analyses of the association between eGFR and recurrence of CRC patients. The *P* value for interaction represents the likelihood of interaction between the variable and the eGFR.

**Figure 3:**
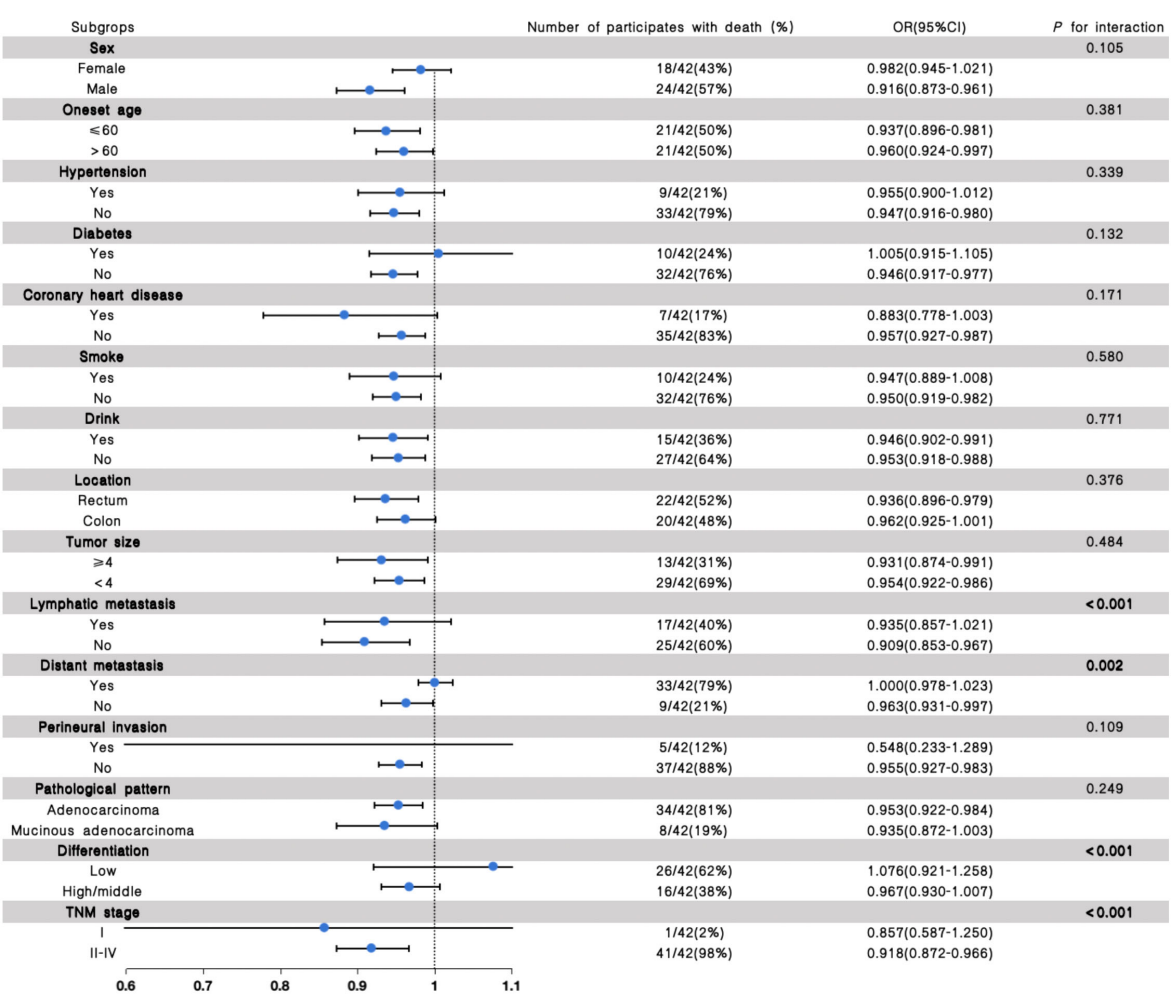
The results of the stratified and interaction analyses of the association between eGFR and prognosis of CRC patients. The *P* value for interaction represents the likelihood of interaction between the variable and the eGFR.

Stratified analyses were further performed to assess the association between eGFR and the outcome of recurrence and clinical prognosis in various subgroups (**Figures 2**, **3**). We further conducted stratified analyses according to subgroups of age, sex, comorbidity smoke, drink, tumor location, size, lymphatic metastasis, distant metastasis, perineural invasion, pathological pattern, tumor differentiation degree, and TNM stage. We found that tumor size (*P* for interactions = 0.020), lymphatic metastasis (*P* for interaction = 0.001), distant metastasis (*P* for interaction <0.001), differentiation (*P* for interaction = 0.001) and TNM stage (*P* for interaction = 0.009) may interact with eGFR in affecting the recurrence of patients with CRC (**Figure 2**), and the lymphatic metastasis (*P* for interaction<0.001), distant metastasis (*P* for interaction = 0.002), differentiation (*P* for interaction<0.001) and TNM stage (*P* for interaction<0.001) may interact with eGFR in affecting the prognosis of patients with CRC (**Figure 3**).

### Differential diagnostic value of eGFR to discriminate between recurrence and no-recurrence, death and survival in patients with CRC

ROC curve analysis indicated that eGFR had the capacity to distinguish recurrence and no-recurrence of patients with CRC with satisfactory sensitivity (60.5%, 95% CI [45.58-73.63]) and specificity (76.0%, 95% CI [62.59-85.70]) (AUC=0.70, 95% CI [0.59-0.81], *P* <0.001) (**Supplementary Figure S2A**, **Supplementary Table S9**). And the eGFR had the capacity to distinguish the survival and death of patients with CRC with satisfactory sensitivity (69.1%, 95% CI [53.97-80.93]) and specificity (74.50%, 95% CI [61.13-84.45]) (AUC=0.74, 95% CI [0.64-0.84], *P* <0.001) (**Supplementary Figure S2B**, **Supplementary Table S10**).

### Analyses of nonlinear relationship between eGFR the outcome of recurrence and prognosis in patients with CRC

**Figure 4:**
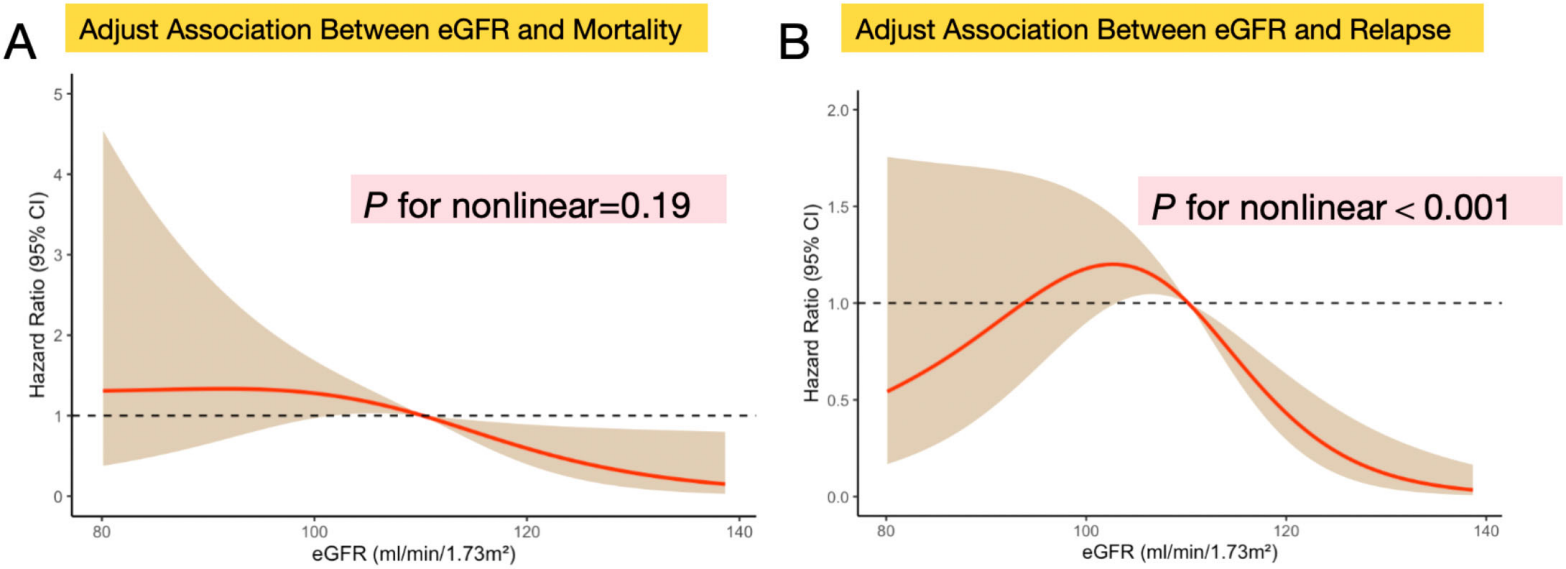
Nonlinear relationship assessment between eGFR levels and clinical prognosis or recurrence in patients with CRC respectively. **(A)** Restricted cubic spline of the association between eGFR and the mortality of CRC patients after adjusting differentiation degree, distant metastasis, lymphatic metastasis, and TNM stage (*P* for nonlinear=0.19); **(B)** Restricted cubic spline of the association between eGFR and the recurrence of CRC patients after adjusting differentiation degree, distant metastasis, lymphatic metastasis, and TNM stage (*P* for nonlinear<0.001);Data were modeled with fixed-effects restricted cubic splines with 3 knots at percentiles 25%, 50% and 75% of the distribution. The light brown shading areas represent the pointwise 95% CI for the fitted nonlinear (red curve). The red curve in the figure illustrates the trend of the adjusted association between eGFR and mortality and recurrence. The eGFR was analyzed as a continuous variable.

Restricted cubic splines showing the association between eGFR and the prognosis and recurrence of patients with CRC are displayed in **Figures 4A, B**. The results show that the relationship between eGFR and prognosis of CRC patients revealed no significant nonlinear correlation (*P* for nonlinear=0.19) after adjusting for potential confounding factors (**Figure 4A**). And the result of smooth curve through the generalized additive model showed that the relationship between eGFR and recurrence of CRC patients was non-linear (*P* for nonlinear<0.001) after adjusting for potential confounding factors (**Figure 4B**).

## Discussion

To our knowledge, this is the first study to evaluate the role of kidney function and the relationship between eGFR and prognosis and recurrence in a moderately - sized sample of newly diagnosed CRC patients. We found that the mean value of eGFR was lower, and the urine specific gravity was significantly higher in patients with CRC compared with HCs, which may indicate the decreased renal filtration and concentrating function in the early stage of CRC patients. In addition, when stratified for eGFR at baseline, a lower eGFR contributes to higher rates of all-cause mortality and recurrence.

In fact, previous studies have shown that there is a significantly increased risk of developing CRC in patients with CKD (13). Moreover, lower eGFR has been associated with higher all-cause and cardiovascular mortality in both vascular disease patients and asymptomatic individuals, which may be due to endothelial dysfunction caused by traditional cardiovascular risk factors and the pro-inflammatory state in CKD (22). Similarly, in our study, we found that the patients with eGFR<90 or 90–110 ml/min had a significantly higher risk of poor clinical prognosis than those with a higher eGFR after adjusting clinical variables, tumor stage, histopathology and other factors of the tumor. Besides, our study found that the lower eGFR often foreshadows a higher rate of recurrence of tumor.

Clearly, further studies are required to understand the molecular mechanisms underlying the link between kidney dysfunction and CRC. In fact, renal insufficiency is a condition where the kidneys’ ability to perform normal functions is reduced, and it has no direct causal relationship with tumor onset (23). Nevertheless, a substantial body of research has indicated that individuals suffering from CKD are confronted with a significantly elevated likelihood of developing tumors (22, 24). This intricate association may be attributed to a multitude of interrelated factors, including alterations in the immune system, persistent states of chronic inflammation, and the progressive accumulation of toxins within the body.

First, chronic inflammation may partially explain the observed association between reduced eGFR and CRC prognosis. Experimental studies indicate that renal dysfunction is associated with elevated pro-inflammatory cytokines (e.g., IL-6) (25), which in preclinical models can enhance tumor angiogenesis and metastatic potential (26). While chronic inflammation is a recognized correlate of both kidney disease and cancer risk in observational studies, the causal direction remains unclear. For instance, systemic inflammation might arise from tumor-derived factors or cancer treatment (27), which could secondarily impair renal function.

Secondly, in the context of renal insufficiency, the reduced excretory capacity of the kidneys may lead to systemic accumulation of uremic toxins, including metabolic waste products and heavy metals (28). Preclinical studies suggest that chronic exposure to these compounds could disrupt cellular homeostasis and induce DNA damage through oxidative stress pathways (29). If operative in humans, such mechanisms theoretically might increase the susceptibility to malignant transformation.

Additionally, preclinical evidence highlights the immune system’s role in surveilling malignant cells (30). In individuals with renal insufficiency, the immune system often becomes dysregulated and functionally impaired (31). This can occur due to a combination of factors, such as uremic toxins interfering with immune cell function, metabolic disturbances associated with kidney disease, and the use of immunosuppressive medications in some cases. A weakened immune system is less effective at detecting and eliminating pre-cancerous or cancerous cells, allowing them to evade immune surveillance and potentially progress into clinically detectable tumors it may affect the prognosis of cancer patients (32). However, the interplay between renal insufficiency, immunity, chronic inflammation, and CRC outcomes remains mechanistically speculative and warrants experimental validation. Besides, our observational design cannot establish whether renal dysfunction directly promotes CRC progression or whether advanced tumor biology itself contributes to kidney injury.

In addition, we found that distant metastasis and low tumor differentiation are more likely to occur in CRC patients with lower eGFR, which may be consistent with the previous literature (33). And eGFR may interact with the pathological features of the tumor, which indicates that renal function and pathological features of tumors may jointly affect the prognosis of patients with CRC. RCS analysis showed that the relationship between eGFR and prognosis of CRC patients had no significant nonlinear correlation, but the relationship between eGFR and recurrence may be non-linear.

Given that renal function is closely related to the prognosis of patients with CRC, it is of utmost importance for CRC patients to engage in the regular monitoring of renal function. Early detection of the changes in renal function may significantly improve treatment outcomes and survival rates. In addition, the determination of kidney function is easy, cost-less, and less invasive, and its values are stable. Moreover, close collaboration with healthcare providers is essential for comprehensive management of the potential risks associated with renal insufficiency. This includes regular medical check-ups, monitoring of kidney function and other relevant biomarkers, and appropriate adjustments to treatment plans as needed. Furthermore, adopting a healthy lifestyle may play a pivotal role in reducing the risk of cancer in this patient population (34).

However, our study contained some limitations. First of all, the retrospective design and the sample size may lead to insufficient statistical power. In addition, this study is based on single-center retrospective data, and the patient population may be influenced by institution-specific diagnostic and treatment norms or regional characteristics, thus introducing sampling bias. Secondly, given that the follow-up duration may not be standardized absolutely, and the time is not long enough, a longer follow-up may contribute to reducing potential bias. Thirdly, the impact of adjuvant therapies on outcomes cannot be ignored, and further investigation is needed to determine the degree and nature of this influence. Fourthly, we have no information on body mass index, nutritional status potential nephrotoxic effects of chemotherapy or muscle mass, although we included clinical diagnoses of comorbidities to exclude these factors. Therefore, in the future, multi - center, large sample, multiple influencing factors, and longer follow-up studies are needed to confirm this view, and animal experiments should be carried out to explore the underlying mechanisms.

## Conclusion

To sum up, this study revealed that CRC patients showed kidney dysfunction, and the eGFR is identified as an independent predictor of disease recurrence and prognosis. Moreover, our study revealed that kidney function has emerged as a potential target for molecular target therapy in patients with CRC.

## Data Availability

The original contributions presented in the study are included in the article/**Supplementary Material**. Further inquiries can be directed to the corresponding author.
