# Supplementary material for: Renal dysfunction as a predictor of recurrence and prognosis in colorectal cancer
Source: Front Oncol. 2025 Jun 27;15:1606286. doi: 10.3389/fonc.2025.1606286 (PMC12245686; doi:10.3389/fonc.2025.1606286)
Supplement: Supplementary file 1 [file DataSheet1.docx]

**Supplementary Material**

**Supplemental** **Methods**

**Figure S-1. CONSORT diagram.** Consolidated Standards of Reporting Trials flow diagram showing study participants screening, eligibility, and inclusion.

**
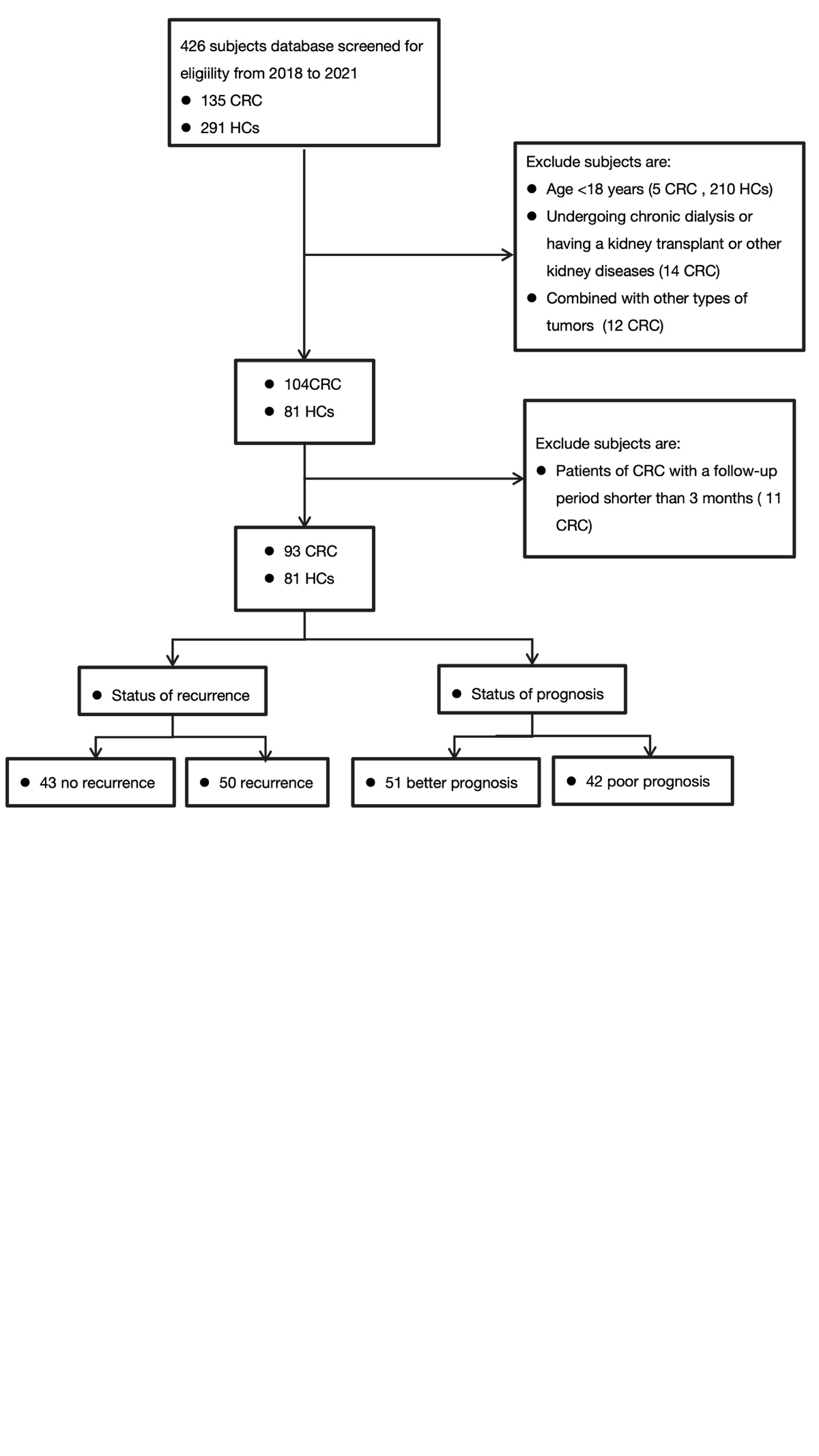
**

**Figure S-1. 1 Consolidated Standards of Reporting Trials flow diagram**

**Supplemental Results**

**Table S-1. The baseline characteristics between included and excluded patients with CRC.**

|  | Included patients | Excluded patients | P value |
| --- | --- | --- | --- |
| Patient numbers | 93 | 42 | - |
| **Clinical characteristics** |  |  |  |
| Female, no. (%) | 36(38.71) | 17(40.48) | 0.846 |
| Onset age, mean (SD), years | 58.45(7.73) | 55.72(15.12) | 0.165 |
| **Comorbidity** |  |  |  |
| Hypertension, no. (%) | 20(21.51) | 7(16.67) | 0.515 |
| Diabetes, no. (%) | 16(17.20) | 7(16.67) | 0.939 |
| Coronary heart disease, no. (%) | 14(15.05) | 6(14.29) | 0.907 |
| Smoke, no. (%) | 24(25.81) | 10(23.81) | 0.805 |
| Drink, no. (%) | 35(37.63) | 15(35.71) | 0.831 |
| Rectum, no. (%) | 50(53.76) | 22(52.38) | ＞0.999 |
| Tumor size＜4cm, no. (%) | 64(68.82) | 28(66.67) | 0.843 |
| Lymphatic metastasis, no. (%) | 22(23.66) | 10(23.81) | ＞0.999 |
| Distant metastasis, no. (%) | 33(35.48) | 14(33.33) | 0.848 |
| Perineural invasion, no. (%) | 10(10.75) | 4(9.52) | ＞0.999 |
| Adenocarcinoma, no. (%) | 76(81.72) | 35(83.33) | ＞0.999 |
| Low differentiation, no. (%) | 29(31.18) | 12(28.57) | 0.841 |
| TNM, I stage, no. (%) | 32(34.41) | 14(33.33) | ＞0.999 |

Abbreviations: SD = standard deviation; IQR = interquartile range; Bold entries indicate *P* < 0.05. TNM = tumor node metastasis classification.

**Table S-2.** **Baseline characteristics of HCs and patients of CRC.**

|  | CRC | HCs | P value |
| --- | --- | --- | --- |
| Patient numbers | 93 | 81 | - |
| **Clinical characteristics** |  |  |  |
| Female, no. (%) | 36(38.71) | 34(41.98) | 0.661 |
| Age, mean (SD), years | 58.45(7.73) | 59.30(6.07) | 0.582 |
| Comorbidity |  |  |  |
| Hypertension, no. (%) | 20(21.51) | 16(19.75) | 0.776 |
| Diabetes, no. (%) | 16(17.20) | 14(17.28) | 0.989 |
| Coronary heart disease, no. (%) | 14(15.05) | 12(14.81) | 0.965 |
| Smoke, no. (%) | 24(25.81) | 21(25.93) | 0.986 |
| Drink, no. (%) | 35(37.63) | 31(38.27) | 0.931 |

Abbreviations: SD = standard deviation; IQR = interquartile range; Bold entries indicate *P* < 0.05. TNM = tumor node metastasis classification.

**Table S-3. Baseline characteristics and kidney function of relapsing and no relapsing CRC patients.**

|  | Relapsing | No relapsing | P value |
| --- | --- | --- | --- |
| **Numbers** | 50 | 43 | - |
| **Clinical characteristics** |  |  |  |
| Female, no. (%) | 17(34.00) | 19(44.19) | 0.315 |
| Onset age, mean (SD), years | 58.27(7.72) | 58.45(7.73) | 0.337 |
| Follow-up time, median (IQR), years | 720(356-976) | 715(356-978) | 0.855 |
| **Comorbidity** |  |  |  |
| Hypertension, no. (%) | 11(22.00) | 9(20.93) | 0.900 |
| Diabetes, no. (%) | 9(18.00) | 7(16.28) | 0.827 |
| Coronary heart disease, no. (%) | 7(14.00) | 7(16.28) | 0.759 |
| Smoke, no. (%) | 14(28.00) | 10(23.26) | 0.602 |
| Drink, no. (%) | 16(32.00) | 19(44.19) | 0.227 |
| Rectum, no. (%) | 31(62.00) | 19(44.19) | 0.086 |
| Lymphatic metastasis, no. (%) | 18(36.00) | 4(9.30) | **0.003** |
| Tumor size＜4cm, no. (%) | 31(62.00) | 33(76.74) | 0.126 |
| Distant metastasis, no. (%) | 26(52.00) | 7(16.28) | **＜0.001** |
| Perineural invasion, no. (%) | 4(8.00) | 6(13.95) | 0.392 |
| Adenocarcinoma, no. (%) | 42(84.00) | 34(79.07) | 0.540 |
| Low differentiation, no. (%) | 23(46.00) | 6(13.95) | **＜0.001** |
| TNM, I stage, no. (%) | 12(24.00) | 20(46.51) | **0.023** |

Abbreviations: SD = standard deviation; IQR = interquartile range; Bold entries indicate *P* < 0.05. TNM = tumor node metastasis classification.

**Table S-4. Increased mortality in lower baseline eGFR categories in CRC patients.**

| **eGFR Strata, mL/min** | **Person-days** | **All-cause death** | **Incidence Rate per 1,000 pd** |
| --- | --- | --- | --- |
| ＜90 | 15142 | 17 | 1.12 |
| 90-110 | 12185 | 12 | 0.98 |
| 110-130 | 23636 | 10 | 0.42 |
| ≥130 | 17623 | 3 | 0.17 |

Abbreviations: eGFR = estimated glomerular filtration rate; pd = person day.

**Table S-5. Baseline characteristics and kidney function of dead and** **survival CRC patients.**

|  | Dead | Survival | P value |
| --- | --- | --- | --- |
| **Numbers** | 42 | 51 | - |
| **Clinical characteristics** |  |  |  |
| Female, no. (%) | 18(42.86) | 18(35.29) | 0.456 |
| Onset age, mean (SD), years | 58.39(7.76) | 58.45(7.73) | 0.739 |
| Follow-up time, median (IQR), years | 720(353-978) | 715(356-978) | 0.295 |
| **Comorbidity** |  |  |  |
| Hypertension, no. (%) | 9(21.43) | 11(21.57) | 0.987 |
| Diabetes, no. (%) | 10(23.81) | 6(11.76) | 0.126 |
| Coronary heart disease, no. (%) | 7(16.67) | 7(13.73) | 0.693 |
| Smoke, no. (%) | 10(23.81) | 14(27.45) | 0.690 |
| Drink, no. (%) | 15(35.71) | 20(39.22) | 0.729 |
| Rectum, no. (%) | 22(52.38) | 28(54.90) | 0.808 |
| Lymphatic metastasis, no. (%) | 17(40.48) | 5(9.80) | **＜0.001** |
| Tumor size＜4cm, no. (%) | 29(69.05) | 35(68.63) | 0.965 |
| Perineural invasion, no. (%) | 5(11.90) | 5(9.80) | 0.745 |
| Distant metastasis, no. (%) | 33(78.57) | 0(0) | **＜0.001** |
| Adenocarcinoma, no. (%) | 34(80.95) | 42(82.35) | 0.862 |
| Low differentiation, no. (%) | 26(61.90) | 3(5.88) | **＜0.001** |
| TNM, I stage, no. (%) | 1(2.48) | 31(60.78) | **＜0.001** |

Abbreviations: SD = standard deviation; IQR = interquartile range; Bold entries indicate *P* < 0.05. TNM = tumor node metastasis classification.

**Table S-6. Univariate and multivariable analysis of factors affecting the prognosis of patients with CRC patients by** **Cox proportional hazards model.**

| Variable | Univariate analysis | | Multivariate analysis | |
| --- | --- | --- | --- | --- |
|  | OR (95%CI) | *P* value | OR (95%CI) | *P* value |
| eGFR | 0.963(0.945-0.981) | **＜0.001** | 0.968(0.946-0.991) | **0.008** |
| Sex | 0.971(0.521-1.809) | 0.926 |  |  |
| Onset age | 0.896(0.484-1.657) | 0.726 |  |  |
| Hypertension | 0.788(0.371-1.670) | 0.534 |  |  |
| Diabetes | 0.680(0.320-1.444) | 0.315 |  |  |
| Coronary heart disease | 0.580(0.251-1.340) | 0.202 |  |  |
| Smoke | 1.668(0.765-3.637) | 1.198 |  |  |
| Drink | 1.136(0.599-2.154) | 0.696 |  |  |
| Location | 0.900(0.487-1.663) | 0.736 |  |  |
| Tumor size | 0.603(0.298-1.221) | 0.160 |  |  |
| Lymphatic metastasis | 0.607(0.317-1.160) | 1.131 |  |  |
| Distant metastasis | 3.344(1.560-7.167) | **0.002** | 15.781(2.835-87.836) | **0.002** |
| Perineural invasion | 0.591(0.226-1.543) | 0.283 |  |  |
| Pathological pattern | 0.775(0.353-1.700) | 0.525 |  |  |
| Differentiation | 2.561(1.346-4.873) | **0.004** | 1.354(0.589-3.112) | 0.475 |
| TNM stage | 2.081(1.418-3.054) | **＜0.001** | 6.443(2.311-17.961) | **＜0.001** |

Abbreviations: eGFR = estimated glomerular filtration rate; OR = Odds ratio; CI = confidence interval; TNM = tumor node metastasis classification; Bold entries indicate *P* < 0.05. eGFR was parameterized as a continuous variable.

**Table S-7. Univariate and multivariable analysis of factors affecting the recurrence of patients with CRC patients by logistic regression models.**

| Variable | Univariate analysis | | Multivariate analysis | |
| --- | --- | --- | --- | --- |
|  | OR (95%CI) | *P* value | OR (95%CI) | *P* value |
| eGFR |  | **0.011** |  | 0.103 |
| ≥130 | REF | - | REF | - |
| 110-130 | 7.265(1.429-36.936) | **0.017** | 7.657(1.335-43.925) | **0.022** |
| 90-110 | 11.375(2.030-63.753) | **0.006** | 8.486(1.322-54.485) | **0.024** |
| ＜90 | 19.500(3.223-117.988) | **0.001** | 10.015(1.230-81.542) | **0.031** |
| Sex | 1.537(0.664-3.558) | 0.316 |  |  |
| Onset age | 2.083(0.909-4.773) | 0.083 |  |  |
| Hypertension | 0.939(0.347-2.535) | 0.900 |  |  |
| Diabetes | 0.886(0.299-2.620) | 0.827 |  |  |
| Coronary heart disease | 1.194(0.383-3.725) | 0.759 |  |  |
| Smoke | 0.779(0.305-1.993) | 0.603 |  |  |
| Drink | 1.682(0.722-3.919) | 0.228 |  |  |
| Location | 2.061(0.899-4.725) | 0.088 |  |  |
| Tumor size | 0.494(0.199-1.227) | 0.129 |  |  |
| Lymphatic metastasis | 5.484(1.685-17.848) | **0.005** | 2.888(0.729-11.443) | 0.131 |
| Distant metastasis | 5.571(2.088-14.867) | **0.001** | 4.343(0.510-36.993) | 0.179 |
| Perineural invasion | 1.865(0.490-7.102) | 0.361 |  |  |
| Pathological pattern | 1.390(0.484-3.988) | 0.541 |  |  |
| Differentiation | 5.253(1.882-4.660) | **0.002** | 1.784(0.409-7.782) | 0.441 |
| TNM stage | 1.852(1.302-2.634) | **0.001** | 0.873(0.388-1.963) | 0.743 |

Abbreviations: eGFR = estimated glomerular filtration rate; REF = Reference; OR = Odds ratio; CI = confidence interval; TNM = tumor node metastasis classification; Bold entries indicate *P* < 0.05. eGFR was parameterized as a categorical variable.

**Table S-8. Univariate and multivariable analysis of factors affecting the recurrence of patients with CRC patients by Poisson regression models**

| **Variable** | **Univariate analysis** | | **Multivariate analysis** | |
| --- | --- | --- | --- | --- |
|  | **OR (95%CI)** | ***P* value** | **OR (95%CI)** | ***P* value** |
| eGFR | 0.968(0.955-0.981) | **＜0.001** | 0.970(0.956-0.984) | **＜0.001** |
| Sex | 1.003(0.632-1.593) | 0.990 |  |  |
| Onset age | 0.930(0.594-1.458) | 0.753 |  |  |
| Hypertension | 0.874(0.515-1.484) | 0.618 |  |  |
| Diabetes | 0.935(0.527-1.661) | 0.819 |  |  |
| Coronary heart disease | 1.122(0.586-2.150) | 0.728 |  |  |
| Smoke | 1.043(0.648-1.680) | 0.861 |  |  |
| Drink | 1.363(0.838-2.217) | 0.212 |  |  |
| Location | 1.132(0.715-1.790) | 0.597 |  |  |
| Tumor size | 0.755(0.486-1.174) | 0.212 |  |  |
| Lymphatic metastasis | 1.636(1.274-2.101) | **＜0.001** | 1.644(1.107-2.442) | **0.014** |
| Distant metastasis | 1.515(1.205-1.906) | **＜0.001** | 1.457(0.525-4.043) | 0.469 |
| Perineural invasion | 2.000(0.845-4.735) | 0.115 |  |  |
| Pathological pattern | 1.007(0.547-1.852) | 0.983 |  |  |
| Differentiation | 1.552(1.217-1.979) | **＜0.001** | 0.880(0.543-1.424) | 0.602 |
| TNM stage | 1.411(1.168-1.705) | **＜0.001** | 1.077(0.712-1.629) | 0.725 |

Abbreviations: eGFR = estimated glomerular filtration rate; OR = Odds ratio; CI = confidence interval; TNM = tumor node metastasis classification; Bold entries indicate *P* < 0.05. eGFR was parameterized as a continuous variable.

**Table S-9.** **Differential diagnostic value of eGFR to discriminate between recurrence and no-recurrence in patients with CRC**

|  | AUC,95%CI | Threshold | LR | 95% CI | Sensitivity | 95% CI | Specificity | *P* |
| --- | --- | --- | --- | --- | --- | --- | --- | --- |
| eGFR | 0.70(0.59-0.81) | 112.3 | 2.519 | 45.58–73.63 | 60.5% | 62.59–85.70 | 76.0% | ＜0.001 |

Abbreviations: eGFR = estimated glomerular filtration rate; CI = confidence interval; AUC = Area under the curve., LR = likelihood ratio

**Table S-10. Differential diagnostic value of eGFR to discriminate between death and survival in patients with CRC**

|  | AUC,95%CI | Threshold | LR | 95% CI | Sensitivity | 95% CI | Specificity | *P* |
| --- | --- | --- | --- | --- | --- | --- | --- | --- |
| eGFR | 0.74(0.64-0.84) | 110.0 | 2.709 | 53.97–80.93 | 69.1% | 61.13–84.45 | 74.5% | ＜0.001 |

Abbreviations: eGFR = estimated glomerular filtration rate; CI = confidence interval; AUC = Area under the curve., LR = likelihood ratio

**
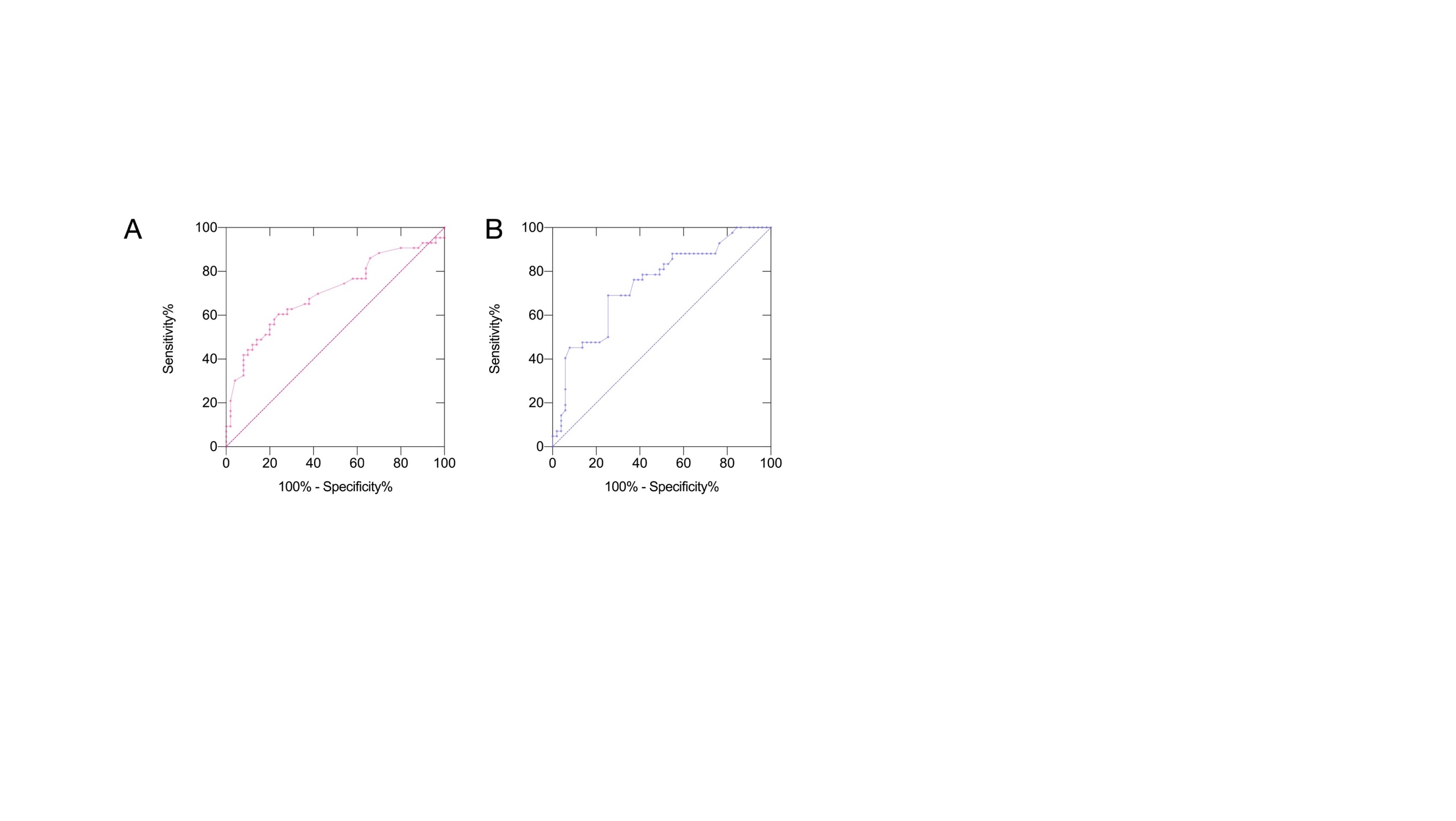
**

**Figure S-2.** **ROC curves of the eGFR for discriminating recurrence and no-recurrence (A); death and survival (B) for patients with CRC.**

**
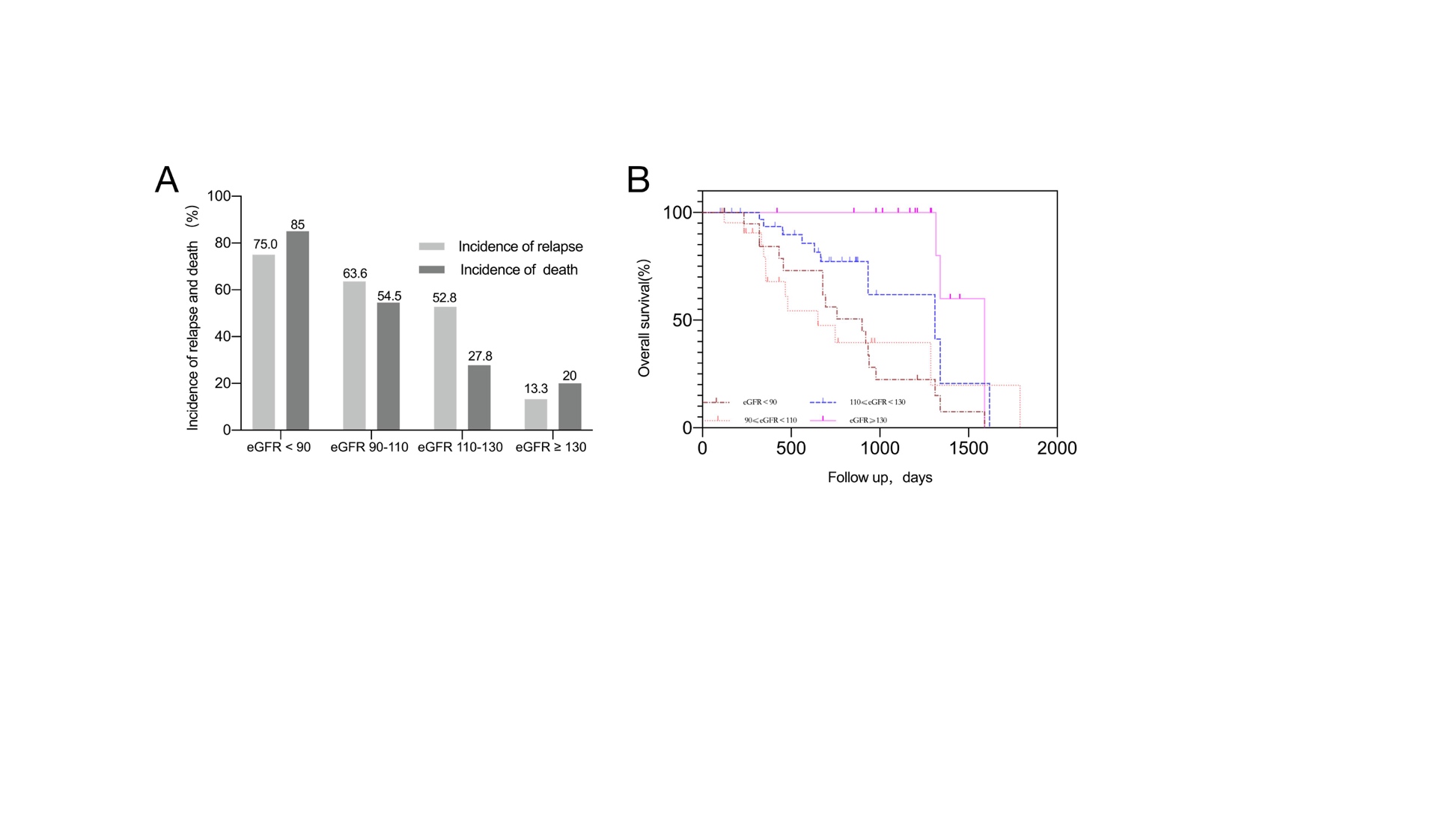
**

**Figure S-3. Incidence of recurrence and death according to eGFR stratification, and Kaplan-Meier survival curves for patients with CRC.** With the increase of eGFR stratification, the recurrence rate and mortality decreased gradually. Light gray represents the incidence of recurrence, dark grey represents the incidence of death (A). The time to death by eGFR stratification as assessed with the Kaplan-Meier estimation. Log-rank (Mantel-Cox) test *P* < 0.001 (B).
